# Bi-Directional Theta Modulation between the Septo-Hippocampal System and the Mammillary Area in Free-Moving Rats

**DOI:** 10.3389/fncir.2017.00062

**Published:** 2017-09-12

**Authors:** Ming Ruan, Calvin K. Young, Neil McNaughton

**Affiliations:** ^1^Department of Psychology and Brain Health Research Centre, University of Otago Dunedin, New Zealand; ^2^Department of Pediatrics and Neonatal Services, Zhuhai Municipal Women’s and Children’s Hospital Guangdong, China

**Keywords:** theta oscillation, hippocampus, septum, supramammillary nucleus, mammillary bodies, granger causality, partial directed coherence, rat

## Abstract

Hippocampal (HPC) theta oscillations have long been linked to various functions of the brain. Many cortical and subcortical areas that also exhibit theta oscillations have been linked to functional circuits with the hippocampus on the basis of coupled activities at theta frequencies. We examine, in freely moving rats, the characteristics of diencephalic theta local field potentials (LFPs) recorded in the supramammillary/mammillary (SuM/MM) areas that are bi-directionally connected to the HPC through the septal complex. Using partial directed coherence (PDC), we find support for previous suggestions that SuM modulates HPC theta at higher frequencies. We find weak separation of SuM and MM by dominant theta frequency recorded locally. Contrary to oscillatory cell activities under anesthesia where SuM is insensitive, but MM is sensitive to medial septal (MS) inactivation, theta LFPs persisted and became indistinguishable after MS-inactivation. However, MS-inactivation attenuated SuM/MM theta power, while increasing the frequency of SuM/MM theta. MS-inactivation also reduced root mean squared power in both HPC and SuM/MM equally, but reduced theta power differentially in the time domain. We provide converging evidence that SuM is preferentially involved in coding HPC theta at higher frequencies, and that the MS-HPC circuit normally imposes a frequency-limiting modulation over the SuM/MM area as suggested by cell-based recordings in anesthetized animals. In addition, we provide evidence that the postulated SuM-MS-HPC-MM circuit is under complex bi-directional control, rather than SuM and MM having roles as unidirectional relays in the network.

## Introduction

Brain oscillations are thought to reflect the organizing of local and global computational units to serve various functions. Rodent hippocampal (HPC) theta rhythmicity has been extensively studied and its behavioral correlates (Buzsáki, 2005), role in modulating local neuronal ensemble activity (Skaggs et al., 1996; Klausberger et al., 2003; Jezek et al., 2011), means of generation (Bland, 1986; Buzsáki, 2002), and propagation (Bland and Oddie, 1998; Pignatelli et al., 2012) are relatively well-understood. Many regions appear to use theta oscillations to temporally coordinate local and long-range excitation-inhibition fluctuations necessary for normal behavioral output (Seidenbecher et al., 2003; Jones and Wilson, 2005; McNaughton et al., 2006; Benchenane et al., 2010; Shirvalkar et al., 2010; Young, 2011; Courtin et al., 2014; Likhtik et al., 2014; Harris and Gordon, 2015).

Diencephalic theta oscillations have mostly been investigated as reflecting the relaying of information pertinent to HPC function (Papez, 1937; Kirk and McNaughton, 1993; Bland and Oddie, 1998; Kirk, 1998; Aggleton and Brown, 1999; Pan and McNaughton, 2004). Under urethane anesthesia, integrity of the supramammillary nucleus (SuM) is crucial for the expression of HPC theta (Kirk and McNaughton, 1991, 1993; Kirk et al., 1996). Given that inactivation rostral to the SuM through to the medial septum (MS) modifies the amplitude but not frequency of reticular-elicited HPC theta oscillations, and that inactivation caudal to the SuM affects both frequency and amplitude, it is believed that SuM transforms tonic input from ascending brainstem regions to a rhythmic input to the MS that is relayed to HPC (Kirk and McNaughton, 1993). SuM would then contribute to behavior via its control of HPC theta oscillations. However, in the freely moving rat, while SuM may control hippocampal theta under some behavioral conditions (Woodnorth and McNaughton, 2002), it has no involvement under others (McNaughton et al., 1995; Thinschmidt et al., 1995). There is also significant downstream modulation of SuM from the HPC, with its most likely function being restricting theta rhythmic activity in the SuM to a lower frequency (Kirk et al., 1996; Kocsis and Kaminski, 2006).

The mammillary nuclei (MM) are thought to be an important relay that facilitates HPC-thalamic coordination in Papez’s circuit (Papez, 1937). In freely moving animals, the majority of single-cell spiking activity in MM is modulated at theta frequencies and appears to contribute to egocentric spatial processing (Blair et al., 1998; Stackman and Taube, 1998; Sharp and Turner-Williams, 2005) via the anterior thalamic nuclei (Bassett et al., 2007) and the postsubiculum (Sharp and Koester, 2008b). Under anesthesia, theta rhythmic bursts in MM appear to be better correlated with ongoing CA1 theta local field potentials (LFPs; Kocsis and Vertes, 1994), and do not survive MS inactivation that abolishes HPC theta LFPs (Kirk et al., 1996). MM thus appears to act as a HPC relay and its rhythmicity could totally depend on the HPC. However, recent data suggest that extra-HPC inputs to the MM may be more important for its functional outputs (Vann, 2013), with the HPC having a modulating role; and, under anesthesia, MM appears to contribute to HPC theta LFPs (Żakowski et al., 2017).

The understanding of information processing and function in the SuM/MM area is an incomplete mosaic. Most SuM/MM recording studies are carried out under anesthesia with only a few examining their functional significance in freely behaving animals (Sharp and Turner-Williams, 2005; McNaughton et al., 2006; Ruan et al., 2011; Hernández-Pérez et al., 2015; Gutiérrez-Guzmán et al., 2017). Given that single-cell and LFP theta oscillations are prominent in the HPC, SuM and MM, and that theta oscillations may reflect functional binding of circuits, we sought to characterize HPC-SuM/MM interactions at theta frequencies. By using bipolar LFP recordings from the HPC and SuM/MM in freely behaving rats, we examined potential distinguishing features in theta LFPs recorded across the SuM/MM area. We applied functional connectivity (coherence and pair-wise phase consistency; PPC) and directional connectivity (partial directed coherence; PDC) measures to gauge the nature of coupled activities in the HPC-SuM/MM circuits. Importantly, we assessed the direction of their modulatory influences. To explicitly examine the dependence of SuM/MM theta LFPs on HPC theta output, we temporarily inactivated the MS to attenuate HPC theta LFPs and characterized the changes in theta LFPs recorded in the SuM/MM. The data obtained from our experiments with freely moving rats reinforce the notion of bi-directional theta modulation between the HPC and SuM/MM. We did not find evidence for MS-inactivation producing divergent neurophysiological changes mirroring single-cell data acquired under urethane anesthesia between SuM and MM. Instead, we report that MS-inactivation reduced the power of SuM/MM theta and increased their dominant theta frequency.

## Materials and Methods

### Subjects

Data from 100 Sprague-Dawley rats from various previous experiments, including some reported previously (McNaughton et al., 2006; Ruan et al., 2011) were pooled for analysis. The final number of rats included in different analyses varied according to Sum/MM recording electrode placement and available behavioral data. These variations are indicated in the “Data Processing” Section and in the “Results” Section. All experiments complied with local ethics committee guidelines (Otago University Animal Ethics Committee: approval numbers 84/00 and 67/03). The rats were obtained from the University of Otago, Department of Laboratory Animal Sciences and kept on a standard 12:12 h light-dark cycle (lights on at 6 am) and received food and water *ad libitum*. After at least 10 days in the laboratory, rats were injected with an anesthetic mix of ketamine/domitor (75 mg/kg @ 100 mg/ml from Parnell Laboratories, New Zealand and 0.5 mg/kg @ 1 mg/ml from Novartis Animal Health, Australia, respectively) for stereotaxic surgery. All procedures were carried out under aseptic conditions and Antisedan (2.5 mg/kg @ 5 mg/ml, Novartis Animal Health, Australia), followed by Temgesic (0.03 mg/kg @ 0.03 mg/ml; or 0.15 mg/rat) were administered for post-operative care. All rats received twisted stainless steel bipolar recording electrode implants in the dorsal HPC (AP −3.8 mm, ML −2.5 mm, DV −3.5 mm, tip separation of ~1.0 mm) and the mammillary area (SuM/MM; @6° angle in the coronal plane, AP −4.8 mm, ML −0.9 mm, DV −9.4 mm, tip separation 0.5 mm) region. In addition, a stainless steel twisted bipolar stimulating electrode (not utilized in the current study) was implanted in the dorsal fornix (@ 8° angle in the coronal plane, AP −1.0 mm, ML −1.0 mm, DV −4.0 mm) and a guide cannula in the MS (26 G, @ 10° angle in the coronal plane, AP 0.2 mm, ML −1.0 mm, DV −5.9 mm). Rats were given 10 days to recover from implant surgery before any behavioral or neurophysiological testing took place.

### Behavioral and Pharmacological Manipulations

To temporarily block HPC theta oscillations, 2% tetracaine (Sigma) in physiological saline (20 mg/ml) was injected into the MS. A syringe pump (Razel Scientific Instruments Inc., Stamford, CT, USA) driving a 10 μL Hamilton syringe connected to the injection cannula (33G) through silicon tubing (50 cm long, ID 0.5 mm, OD 2.2 mm; Cole-Parmer Instrument Company, Vernon Hills, IL, USA) was used to deliver 0.5 μl tetracaine at 0.5 μ/min.

All behavioral procedures were as described in more detail in previous studies (McNaughton et al., 2006; Ruan et al., 2011). The tetracaine data analyzed here were extracted from the time at which freely behaving rats were tethered to the recording system, up until a few minutes after visual confirmation of HPC theta LFPs have been attenuated. In the OF condition (Ruan et al., 2011), rats were left to explore a square box (73 × 73 × 51 cm) for at least 6 min.

### Anatomical Reconstruction

**Figure 1:**
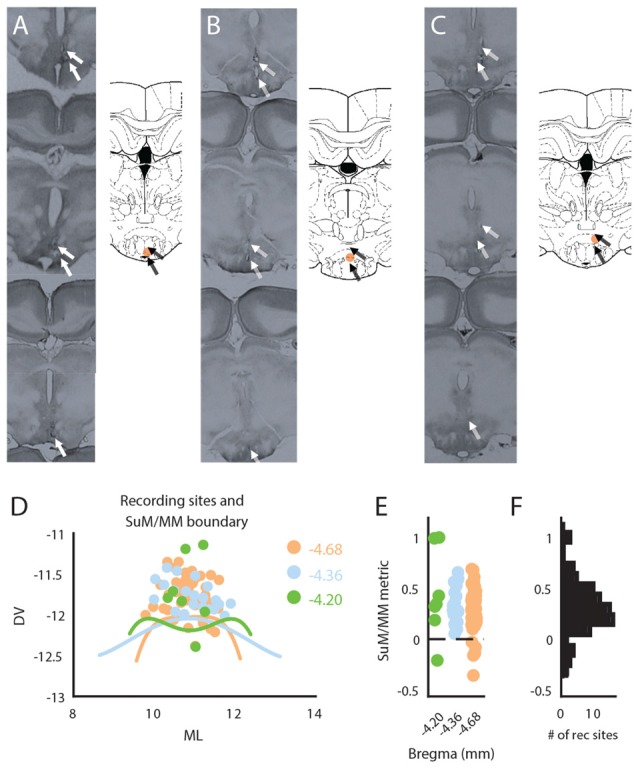
Anatomical reconstruction and distribution of recording sites within the mammillary (MM) area. **(A)** Raw histological sections (left) and reconstructed recording site (right) in the MM. **(B)** Raw histological sections (left) and reconstructed recording site (right) in the SuM/MM border. **(C)** Raw histological sections (left) and reconstructed recording site (right) in the SuM. **(D)** Anatomical boundaries between the SuM and MM with putative recording sites across three anterior-posterior positions according to the brain atlas. **(E)** Distribution of recording sites as per our SuM/MM metric. **(F)** Total distribution of recording sites across our SuM/MM metric. DV, Dorso-ventral; ML, Medio-lateral; SuM, supramammillary nucleus; MM, mammillary bodies.

At the conclusion of experiments, rats were deeply anesthetized with sodium pentobarbital and transcardially perfused with physiological saline followed by 10% formalin. After at least 24 h in 10% formalin, the brains were cryoprotected with 30% sucrose solution in 10% formalin prior to sectioning (90 μm) on a freezing microtome. After histological reconstruction of bipolar wire tips in brain sections, the mid-distance between the tips was designated as the “recording site” (e.g., Figures 1A–C). This was done to better reflect the presumed anatomical source of recorded LFPs between the bipolar electrode pair. For SuM/MM recording sites, the anatomical boundaries across three consecutive coronal planes between the SuM and MM were approximated by fitting a 4th order polynomial (Figure 1D) over sample points taken from the boundaries indicated on the Paxinos and Watson brain atlas (Paxinos and Watson, 2007). Locations of recording sites, calculated as the orthogonal distance to the boundary lines, were taken as a metric to indicate relative position to the SuM/MM boundary. In this scheme, positive values essentially represent more dorsal (SuM) recording sites and negative values represent ventral (MM) recording sites, with zero values being sites on the boundary between the two. This metric was used to provide a continuous scale to locate SuM and MM LFPs.

In addition, the guide cannula targeting the MS was also identified by the deepest track identified on serial 90 μm sections. We assumed the spread of tetracaine to be a 1 mm sphere, consistent with the spread of dyes (Myers, 1966; McNaughton, 1977) at 0.5 μl.

### Data Processing

LFPs were acquired by a Micro1401 (CED, UK) at a sampling rate of 100 Hz after being passed through unity-gain pre-amplifiers and Grass P511K amplifiers. All data were exported to Matlab (Mathworks, Natick, MA, USA) for further analysis at a re-sampled frequency of 128 Hz using in-built cubic spline interpolation in Spike2 (CED, UK). Hardware gain was set differently for different sessions and rats. All raw LFPs were *z*-scored for each individual session prior to further analyses.

Not all rats were subjected to all behavioral contexts (OF, tetracaine block) for testing; therefore, data selection on the basis of behavioral context overlap in different recording sessions was made. Particularly, a total of 68 rats with correct HPC/SuM/MM electrode placements contributed to the study, with all rats contributing to OF sessions and 46 to tetracaine block (note three were later excluded for a lack of statistically significant demonstration of HPC theta power decrease from MS inactivation).

### Frequency Analyses

Theta frequency range in the current study is defined as 5–12 Hz. All time-frequency analyses were done with a 2 s window with a 90% overlap. The cutoff frequency for all analyses is 50 Hz. All spectral analyses were carried out using the multi-taper method, with three tapers and a numerical bandwidth product of 5 using the Chronux package (Bokil et al., 2010). Percentage theta power is derived from the proportion of 5–12 Hz power in the full spectrum (1–50 Hz).

### Partial Directed Coherence

To estimate the direction of theta rhythmic modulation between the HPC and SuM/MM, we performed PDC calculations (Sameshima and Baccalá, 1999; Gourévitch et al., 2006; Young and Eggermont, 2009). Briefly, a step-wise least-squares estimation of optimal model order for non-stationary LFP data was calculated for fitting an auto-regressive model to the data by applying the Bayesian information criterion. Multivariate auto-regressive model coefficients were calculated using the optimal model order generated above and segments of 2 s windowed data. The MVAR model coefficients were then transformed into the frequency domain, subtracted from the identity matrix and normalized to yield an estimation of “causal influence” between HPC and SuM/MM.

For time-reversed PDC (Winkler et al., 2016), the individual windowed segments were subjected to time-reversal; that is, the same 2 s windowed data points were used for time-forward and reversed PDC. The model order generated by the initial step-wise least-squares estimation for forward-time whole session data was retained for calculating the MVAR model coefficients for time-reversed PDC as well. To examine the symmetry of time-forward and reversed PDC scores, a difference ratio is calculated between HPC->SuM/MM and SuM/MM->HPC under the same direction in time, and the difference between time-forward ratio and time-reversed ratio was taken to indicate a change in PDC symmetry. A large score (asymmetric estimates) reflects true underlying “causal influence” while a low score (symmetric estimates) suggests PDC estimates may be biased by low signal-to-noise ratio (SNR) of input LFPs.

To estimate the overall direction of “causal influence”, the difference between HPC->SuM/MM and SuM/MM->HPC PDC was used as an estimator. This signed score is positive if HPC theta activity “Granger causes” SuM/MM theta activity, and vice versa if it is negative. The score itself reflects the estimated magnitude of the relationship.

### Granger Causality

To estimate the source of induced changes in tetracaine time-course data, we calculated the amount of “causal flow” (Seth, 2005) between rates of change before, during and after tetracaine administration. The model order for each analysis is determined independently as the average model order necessary to account for data complexity (Bayesian information criterion) from each rat, for each pair-wise comparison. F-ratios generated by regression models were taken as the magnitude of “causal flow”. The amount of in/out “causal flow” for each rate of change was calculated as the sum of statistically significant (*p* < 0.05, Bonferroni corrected) F-ratios; that is, F-ratios with corrected *p* > 0.05 are set to zero, see Seth (2005). A predictive power score was calculated as the difference between in/out “causal flow” estimates for each rate of change.

### Dominant Frequency as Center of Mass

As a measure of dominant frequency in the theta band, we used center of mass (CoM) of the area under the theta band. Specifically, we calculated the weighted mean of theta band power from 5 Hz to 12 Hz in 0.5 Hz bins.

### RMS

The root mean squared amplitude of LFP signals was calculated as a 4 s moving average of 100 ms windowed RMS.

### Pair-Wise Phase Consistency

Phase angles from Hilbert-transformed data were used to calculate the distribution of relative angular distances between HPC and SuM/MM phases to derive an estimate of phase coupling that is free of sample bias (Vinck et al., 2010).

### Time-Domain Analysis

To gauge the dynamic changes of signal RMS values, theta power spectral density (PSD) and PDC across time, we extracted data from several minutes before tetracaine injection to the MS, until HPC theta blockade was visually judged to have taken place. During this period, rats were placed in a cylindrical enclosure (30 cm in diameter, 30 cm tall) to which they had been exposed previously for verifying the quality of the recordings and the effectiveness of stimulation. Rats were picked up once to replace the dummy cannula with the injection cannula, otherwise they were allowed to freely behave. These data were then aligned by taking the point where a reduction of 50% HPC LFP RMS normalized by individual session RMS ranges from the start of recording to the start of further manipulations (McNaughton et al., 2006). All data were extracted to have equal-length, based on the minimum length available to and from the 50% RMS reduction point; this was approximately 500 s before the reduction point and 180 s after it. To compare pre- and post-MS inactivation effects, we further extracted ± 180 s of data centered around the 50% RMS point.

## Results

A total of 68 rats were identified to have their recording electrodes placed at the level of SuM/MM. Representative raw histology, and reconstructed bipolar tips found in the MM (Figure 1A), SuM/MM overlapping (Figure 1B) and SuM (Figure 1C). A 2D distribution of the designated recording sites collapsed across various anterior-posterior points considered in the current study is presented in Figure 1D. The distribution of placements at the three coronal planes is presented in Figure 1E using our metric where zero marks the anatomical border between SuM and MM between all three planes. While the total number of placements (as the mid-distance between the two poles of the recording electrode) in the SuM predominates, most of the placements were found to be close to the atlas-derived border between MM and SuM (Figure 1F) and virtually all the MM recording sites come from more caudal placements (Figure 1E).

### Theta Field Oscillations in the SuM/MM Area during Free Behavior

**Figure 2:**
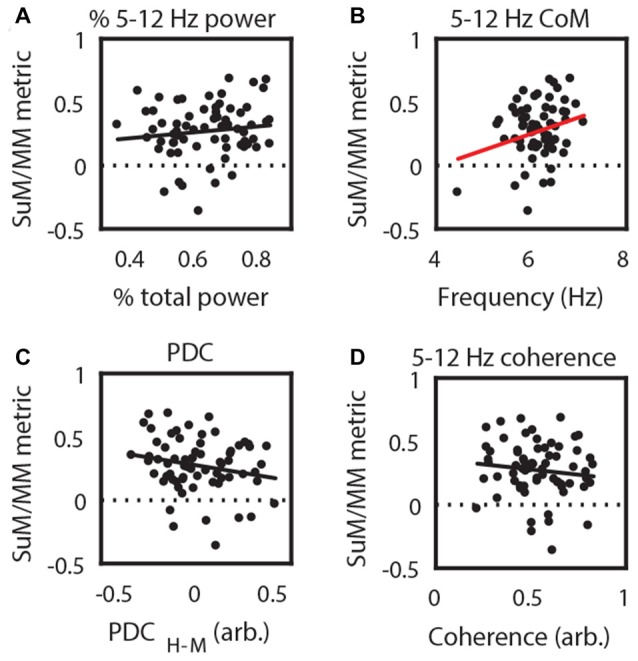
Correlating recording position with theta local field potential (LFP) characteristics. **(A)** 5–12 Hz power; **(B)** 5–12 Hz CoM as a measure of dominant frequency; **(C)** 5–12 Hz coherence; and **(D)** 5–12 Hz PDC from SuM/MM recording sites. Red regression line denotes statistically significant correlations. CoM, center of mass; PDC, partial directed coherence; H-M, Hippocampal (HPC)-SuM/MM PDC difference score; SuM, supramammillary nucleus; MM, mammillary bodies.

Theta rhythmic bursting and LFPs in the SuM/MM are affected by MS inactivation (Kirk et al., 1996; McNaughton et al., 2006) in a way that suggests that MM theta activities are dependent on input from the HPC, while SuM may receive or generate some theta rhythmicity independent of HPC. We sought to identify the relationship between theta LFPs and recording location within the SuM/MM area in freely moving rats using data from the OF condition. There was no significant correlation between SuM/MM theta power and SuM/MM distance metric (*r* = 0.13, *p* = 0.31; Figure 2A), while there was a weak positive correlation between SuM/MM metric and the dominant theta frequency (*r* = 0.26, *p* < 0.04; Figure 2B); that is, the predominant theta frequency is higher for recordings that are closer to the SuM than the MM. No significant correlations exist between the SuM/MM metric and theta coherence (*r* = −0.12, *p* = 0.33; Figure 2C) but a non-significant inverse relationship was observed between SuM/MM metric and extent of HPC theta “causal flow” (PDC) to the area (*r* = −0.21, *p* = 0.09; Figure 2D), suggesting more dorsal recordings (more affected by SuM) may have marginally better predictive power over HPC theta, whereas more ventral (more affect by MM) recordings are better predicted by HPC theta.

**Figure 3:**
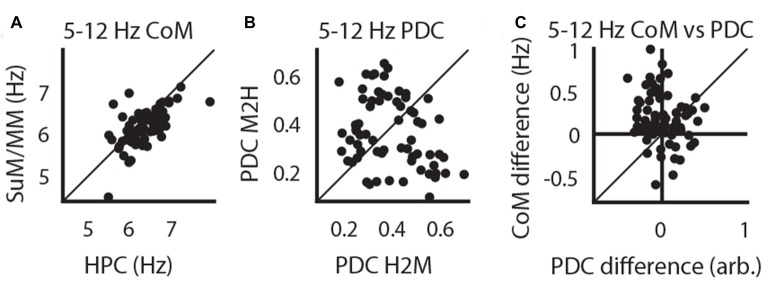
Higher HPC theta frequency is related to a higher SuM/MM to HPC drive. **(A)** Paired comparisons of dominant HPC and SuM/MM theta frequency. **(B)** Paired comparisons between theta PDC estimates for HPC to SuM/MM (H2M) and the opposite direction (M2H). **(C)** Difference between HPC and SuM/MM dominant theta frequency is plotted against the difference between H2M and M2H PDC estimates (i.e., overall causal influence). CoM, center of mass; PDC, partial directed coherence; H2M, mean HPC->SuM/MM theta PDC; M2H SuM/MM->HPC theta PDC; H-M, HPC-SuM/MM PDC difference score; SuM, supramammillary nucleus; MM, mammillary bodies.

Previous research indicates that both the SuM (McNaughton et al., 1995; Kocsis and Kaminski, 2006) and the MM (Sharp and Koester, 2008a) may be involved in driving HPC theta LFPs at higher frequencies. To address this issue with LFP recordings, we asked if there is evidence that HPC and SuM/MM operate at different frequencies, and whether PDC is frequency dependent. Theta power CoM analysis revealed that across pairs of recordings, dominant theta frequency found in HPC tends to be higher than SuM/MM theta (*t*_(66)_ = 3.34, *p* < 0.01; Figure 3A). To specifically ask if this difference in dominant frequency between SuM/MM and HPC can be related to the direction of theta propagation, we further investigated the relationship between directional PDC scores. Firstly, there is no overall bias in HPC to SuM/MM (H2M) and SuM/MM to HPC (M2H) PDC scores (*t*_(66)_ = 0.76, *p* = 0.45; Figure 3B), with a mixture of both directional estimates. To specifically relate ongoing theta frequency and effective connectivity, we examined the relationship between the difference of HPC and SuM/MM CoM from ordinary spectra, against the difference between the magnitudes of directional flow from PDC. In Figure 3C, data points are clustered in the top left quadrant where higher HPC frequency (positive value on the *y*-axis) is linked to a SuM/MM to HPC direction of theta propagation (negative value on the *x*-axis).

### MS Inactivation Reveals Partial Dependence of SuM and MM Theta LFPs on the MS-HPC System

**Figure 4:**
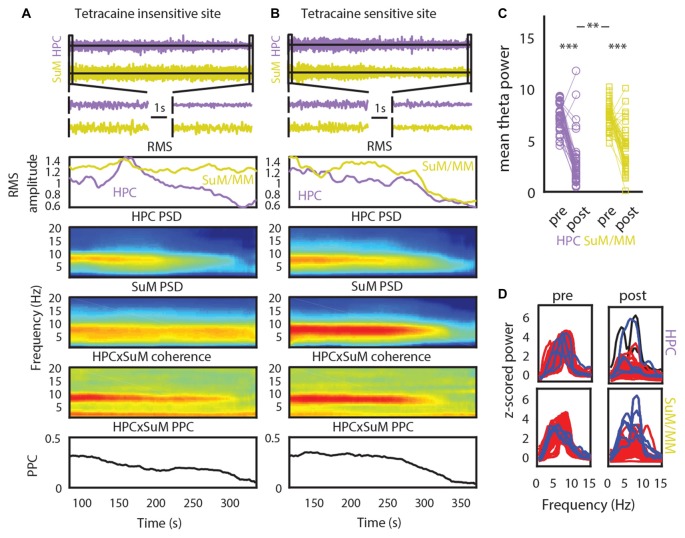
The effect of tetracaine injection to the medial septal (MS). **(A)** An example of a MS-inactivation insensitive site where SuM theta PSD persists throughout the recording. Top to bottom panels: change in HPC and SuM raw LFP, expanded raw LFP segments, RMS, HPC PSD, SuM PSD, HPC-SuM coherence and HPC-SuM theta PPC **(B)** same as in **(A)** but with a SuM site that demonstrated sensitivity to MS-inactivation. **(C)** Theta PSD changes as a result of MS-inactivation across HPC and SuM/MM from all sessions. **(D)** Raw spectra show data from all recording pairs, with those that showed a statistically significant decrease (red traces) of HPC theta PSD attenuation, those did not (black traces) and those with a statistically significant increase (blue traces) of SuM/MM theta PSD after MS-inactivation. RMS, root mean squared power; PSD, power spectral density; PPC, pair-wise phase consistency; HPC, hippocampus; SuM, supramammillary nucleus; MM, mammillary bodies. **p < 0.01, ***p < 0.001.

Our initial analysis of freely moving LFPs corroborates previous findings that SuM theta oscillations may contribute to the generation of higher frequency theta oscillations in the HPC (Kocsis and Kaminski, 2006). As MS-inactivation abolishes single-cell MM rhythmic bursts and increases SuM bursting frequency (Kirk et al., 1996), we sought to investigate the modification of LFP rhythmic activities in the SuM/MM area after reducing HPC theta by inactivating the MS. Data collected from a holding box before and during tetracaine infusion, and prior to further testing as part of a different study (McNaughton et al., 2006) was used for analysis (see “Time-Domain Analysis” in “Materials and Methods” Section). Figures 4A,B illustrate the time-course of raw LFP, LFP RMS, PSDs, coherence and theta PPC around the time of injection for recording sites that appear to be resistant to MS inactivation (Figure 4A) or sensitive to it (Figure 4B). As can be seen in both panels, large decreases in RMS, theta PSD, theta coherence and theta PPC occur concurrently. In the tetracaine insensitive case (Figure 4A), SuM/MM theta power is seen to continue throughout the record, in comparison to where tetracaine application also attenuates SuM/MM theta PSD (Figure 4B). However, the predominant response to MS inactivation by tetracaine is a reduction of theta PSD in both HPC and SuM/MM (Figure 4D), where statistically significant decreases in theta PSD occurs in the HPC (*t*_(43)_ = 11.75, *p* < 0.001) and SuM/MM (*t*_(43)_ = 7.79, *p* < 0.001). There is also a statistically significant difference between the magnitude of theta PSD decrease between HPC and SuM elicited by MS inactivation (*t*_(43)_ = 2.79, *p* < 0.008), indicating tetracaine attenuated theta PSD more at HPC recording sites than SuM/MM sites. Data presented in Figure 4C as raw spectra (Figure 4D) indicate that the most common response to MS-inactivation is HPC theta power reduction (red traces); only a single case where an increase of theta power was observed in the SuM/MM (blue traces) is associated with ineffective MS-inactivation, as indicated by high theta power after inactivation (Figure 4D, top right). There are also a few instances where the perseverance of theta activity in the SuM/MM shifted to a higher frequency (Figure 4D, blue traces, bottom right).

**Figure 5:**
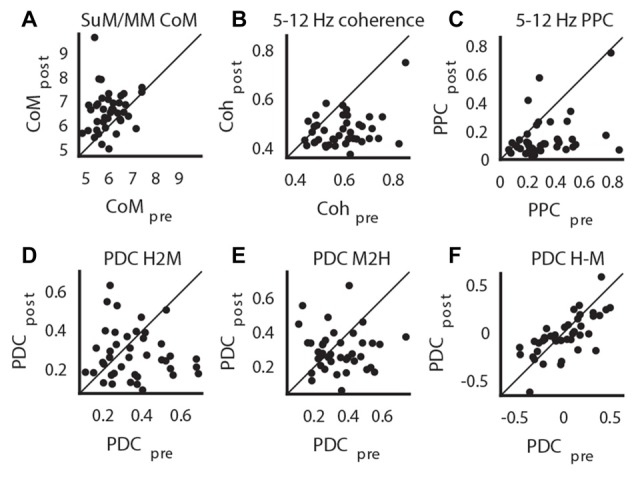
Impact of MS-inactivation in other measures of theta LFP profiles. **(A)** Change in SuM/MM dominant theta frequency. **(B)** Change in theta coherence between HPC and SuM/MM. **(C)** Change in theta PPC between HPC and SuM/MM. **(D)** Change in H2M theta PDC. **(E)** Change in M2H theta PDC. **(F)** Change in H-M theta PDC. CoM, center of mass; PDC, partial directed coherence; PPC; pair-wise phase consistency; H2M, mean HPC->SuM/MM theta PDC; M2H SuM/MM->HPC theta PDC; H-M, HPC-SuM/MM PDC difference score; SuM, supramammillary nucleus; MM, mammillary bodies.

We applied a criterion where only statistically significant HPC theta PSD decreases within each case are retained for subsequent analyses to limit the analyses to those cases where there is clear MS inactivation as measured by the attenuation of HPC theta PSD. The significance level was Bonferroni corrected at alpha = 0.01. With this criterion, one case of no statistically significant effects of MS inactivation on HPC theta PSD and two cases of HPC theta PSD increase after MS inactivation were excluded. Corroborating the raw spectra in Figure 4D, we found a significant increase of dominant theta frequency in the SuM/MM post-MS inactivation (*t*_(40)_ = 4.07, *p* < 0.001; Figure 5A). Both theta coherence and PPC are consistently reduced by MS inactivation (*t*_(40)_ = 7.14, *p* < 0.001; Figure 5B, *t*_(40)_ = 5.33, *p* < 0.001; Figure 5C, respectively). The effect on PDC measures were as expected, where MS inactivation mostly decreased HPC->SuM/MM drive (*t*_(40)_ = 2.29, *p* < 0.03; Figure 5D), whereas no statistically significant change was detected for PDC estimates in the other direction (*t*_(40)_ = 1.65, *p* = 0.10; Figure 5E). Interestingly, despite the significant decrease of HPC->SuM/MM PDC scores, PDC difference scores were not found to be statistically significant (*t*_(40)_ = 1.15, *p* = 0.26; Figure 5F), suggesting no changes in the predominant pattern of SuM/MM->HPC drive pre- and post-MS-inactivation.

The removal of theta rhythmic HPC input may further unmask dissociating features of SuM/MM theta LFPs (Kirk et al., 1996). We correlated the magnitude of change in theta PSD to the SuM/MM metric to see if the sensitivity to MS inactivation at the SuM/MM recording site can be used to distinguish the localization within the area. We found no evidence for discrete SuM/MM differences according to tetracaine sensitivity based on changes in theta PSD caused by MS-inactivation (Tables 1, 2, SuM/MM PSD). Since we found a weak relationship between dominant theta frequency in the SuM/MM and the SuM/MM distance metric in free behavior (see Figure 2B), we then searched for any changes relating to theta PSD caused by MS inactivation that may provide some predicative power to SuM/MM localization (Tables 1, 2). None of the measures we examined relating to local SuM/MM theta, measures of functional or directional connectivity with the HPC revealed any significant correlations. Notably, theta PDC and CoM, which were found to weakly predict recording location (Figures 2B,D), yielded the highest correlation coefficients and smallest *p*-values in our correlation analyses. These data collectively suggest MS-inactivation cannot be used to differentiate the SuM/MM area as used in single-cell recordings in anesthetized rats. In fact, our data suggest that the removal of putative HPC theta rhythmic input renders SuM/MM theta activities more similar in the freely moving rat.

**Table 1:** Correlations and their *p*-values between the supramammillary/mammillary (SuM/MM) metric and the change (post-pre) of theta-related measures in the SuM/MM, induced by medial septal (MS) inactivation.

Variable	*r*	*p*-values
SuM/MM CoM_post-pre_	−0.21	0.1517
PDC M2H_post-pre_	−0.13	0.4077
PDC H-M_post-pre_	0.12	0.4435
PDC H2M_post-pre_	0.05	0.7234
5–12 Hz PPC_post-pre_	−0.05	0.7425
SuM/MM PSD_post-pre_	−0.03	0.8531
PDC M2H CoM_post-pre_	0.03	0.8574
5–12 Hz coherence_post-pre_	0.02	0.8766

**Table 2:** Correlations and their *p*-values between the SuM/MM metric and theta-related measures post- MS inactivation in the SuM/MM.

Variable	*r*	*p-values*
SuM/MM CoM_post_	−0.16	0.2785
PDC H2M_post_	−0.08	0.5948
PDC H-M_post_	−0.07	0.6644
SuM/MM PSD_post_	−0.06	0.7094
5–12 Hz PPC_post_	−0.05	0.7633
5–12 Hz coherence_post_	−0.04	0.7676
PDC M2H_post_	0.03	0.8263
PDC M2H CoM_post_	−0.01	0.9559

**Figure 6:**
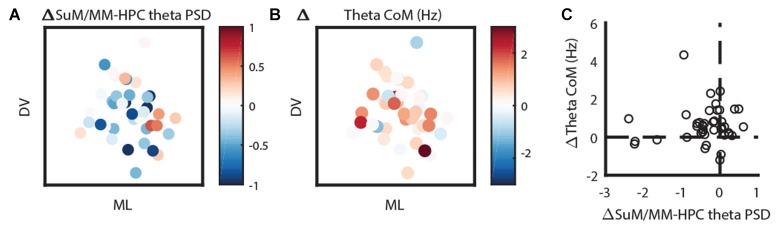
Two-dimensional localization of theta-related changes in the SuM/MM area. **(A)** The change of SuM/MM theta power relative to HPC changes in a coronal plane collapsed through three anterior-posterior planes. Warmer colors indicate more “sensitivity” (i.e., relatively more SuM/MM theta power decrease compared to HPC) to MS-inactivation. **(B)** Same layout as **(A)**, but colors indicate the magnitude and direction of dominant theta frequency changes seen in the SuM/MM comparing post- and pre-MS-inactivation. **(C)** Correlating relative SuM/MM theta power changes to the HPC and the change in dominant SuM/MM theta frequency after MS-inactivation. HPC, hippocampus; SuM, supramammillary nucleus; MM, mammillary bodies; ML, medio-lateral; DV, dorso-ventral.

Given the most striking findings from our MS-inactivation experiments are SuM/MM theta power attenuation with an increase of dominant theta frequency, we mapped these changes back to their estimated anatomical sources. Figure 6A shows the change of SuM/MM theta power before and after MS-inactivation relative to simultaneously recorded HPC theta power. As described earlier, SuM/MM power changes are largely modest compared to associated HPC changes (values <0), with a “pocket” of exceptions where SuM/MM theta power reduction were found to be more drastic than HPC theta power reductions (values >0) on the left lateral aspects of the SuM. The change of dominant theta frequency in the SuM/MM area has a more uniform distribution, but a pattern can be seen that most of the higher frequency increases appear to be at the level of SuM close to the MM border, with a single MM recording site showing the largest change (Figure 6B). To examine if these two qualities are correlated, we plotted the relative change of SuM/MM theta power and the change of SuM/MM theta frequency in Figure 6C. We found no correlation between the two variables.

**Figure 7:**
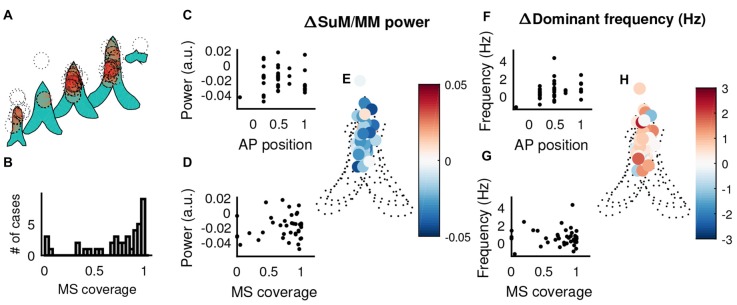
The influence of MS cannula placement in theta-related changes elicited by MS-inactivation. **(A)** Liberal estimates of tetracaine spread (1 mm diameter centered on reconstructed cannula tip, dotted circles) and affected areas within the MS (shaded in red) in the coronal plane, across different points on the anterior-posterior axis. **(B)** Distribution of MS coverage of the 1 mm “circle of influence”. **(C)** Correlation between the relative change of SuM/MM theta power normalized by HPC theta power change and the recording position in the anterior-posterior axis. **(D)** Correlation between the relative change of SuM/MM theta power normalized by HPC theta power change and the estimated on/off target effects. **(E)** The change of SuM/MM theta power relative to HPC changes in a coronal plane collapsed through five anterior-posterior planes around the septal region. Warmer colors indicate more “sensitivity” (i.e., relatively more SuM/MM theta power decrease compared to HPC) to MS-inactivation. **(F)** Correlation between the change of SuM/MM dominant theta frequency and the recording position in the anterior-posterior axis. **(G)** Correlation between the change of SuM/MM dominant theta frequency and the estimated on/off target effects. **(H)** Changes in SuM/MM theta dominant frequency in response to MS-inactivation mapped in coronal planes across five anterior-posterior points. Colors indicate the magnitude and direction of dominant theta frequency changes seen in the SuM/MM comparing post- and pre-MS-inactivation. HPC, hippocampus; MS, medial septum; SuM, supramammillary nucleus; MM, mammillary bodies; AP anterior-posterior.

Given there does not appear to be a relationship between SuM/MM recording sites and our measures of theta oscillation properties, we examined the potential for MS cannula placement to contribute to the patterns of power and frequency changes observed in the SuM/MM. We assume a uniform, maximal diffusion radius of 1 mm for 0.5 μl injections of tetracaine based on previous studies and our own experience (Myers, 1966; McNaughton, 1977). The estimated injection spread in the coronal plane from all reconstructed cannula tracks (Figure 7A) suggests the majority of the MS-inactivations were centered on the MS with a few exceptions. There were four cases where injections were essentially made in the LS/septohippocampal nucleus without potentially affecting the MS (Figure 7B). First, we examined the relationship between SuM/MM theta power change upon MS-inactivation (i.e., difference between post- and pre-inactivation theta power) and the placement of the guide cannula in the anterior-posterior axis (Figure 7C) but found no significant correlation (*r* = −0.02, *p* = 0.92). The change in SuM/MM theta power was also not related to the amount of estimated MS coverage of our injections (Figure 7D; *r* = 0.11, *p* = 0.50). Cannula placements in the coronal plane also fails to show any discernible pattern in their role to systematically influence the relative change of theta power between the SuM/MM and HPC (Figure 7E). There is a weak tendency for more rostral sites to elicit an increase of dominant frequency (Figure 7F; *r* = 0.33, *p* = 0.03). The coverage of MS (inversely, the potential off-target effects) did not correlate with a change in SuM/MM dominant frequency (Figure 7G; *r* = −0.06, *p* = 0.73). Again, mapping the magnitude and direction of SuM/MM theta frequency change in the coronal plane of reconstructed cannula sites did not reveal any patterns (Figure 7H).

### Reduction of HPC Theta Power as a Source of Artifactual PDC Estimation

**Figure 8:**
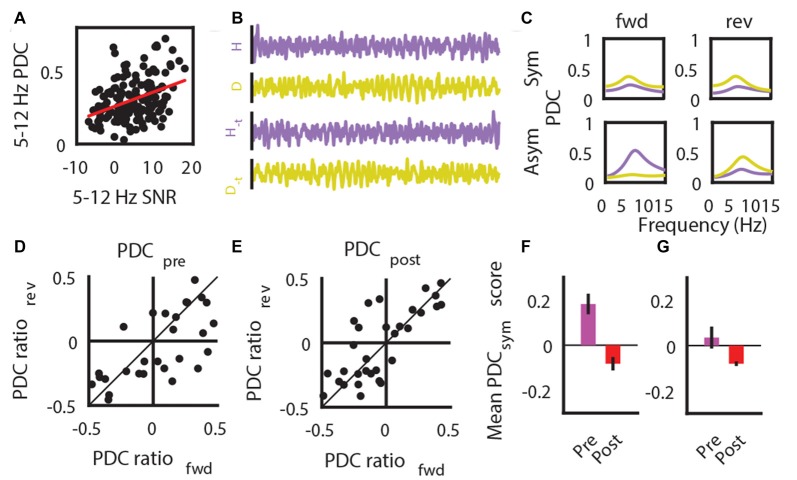
Low SNR impacts on PDC estimates. **(A)** SNR correlation with the magnitude of PDC estimates. **(B)** Example LFP traces that are in its original form and time-reversed form (_*−t*_). **(C)** Example data showing results comparing the magnitude of forward (fwd) and reversed (rev) time PDC measures results in symmetric estimates (top row) indicative of erroneous estimates of causal influence due to low SNR, and asymmetric (bottom row) estimates that is consistent with expected outcome of time-reversal. **(D)** PDC symmetry between forward (fwd)- and reversed (rev)- time estimates, as assessed by a difference ratio between HPC to SuM/MM PDC and SuM/MM to HPC PDC estimates prior to MS-inactivation. **(E)** PDC symmetry between forward (fwd)- and reversed (rev)- time estimates, as assessed by a difference ratio between HPC to SuM/MM PDC and SuM/MM to HPC PDC estimates after MS-inactivation. **(F)** Mean magnitude of overall HPC-SuM/MM PDC differences pre- and post-MS-inactivation, based on data points that have significantly asymmetric PDC for fwd- or rev time estimates. **(G)** Mean magnitude of overall HPC-SuM/MM PDC differences pre- and post-MS-inactivation, based on data points that have significantly asymmetric PDC for fwd- and rev- time estimates. PDC, partial directed coherence; SNR, signal-to-noise ratio; H, hippocampal LFP; M, mammillary area LFP; _*−t*_, time-reversed LFPs; Sym, symmetrical PDC spectra; Asym, asymmetrical PDC spectra; PDC_sym_, PDC symmetry score.

It is known that SNR plays a role in the utilization of auto-regressive modeling based algorithms to estimate the direction of information flow (Bastos and Schoffelen, 2015; Winkler et al., 2016). Our findings indicate that, despite a presumed decrease of HPC->SuM/MM directional flow of theta oscillations, there was no change in overall directionality of theta propagation. This was most likely due to the non-statistically significant decrease of the SuM/MM->HPC contribution to the system (Figure 5E), corroborating an overall decreased SuM/MM theta PSD reported above (Figure 4C). However, our manipulation literally abolishes HPC theta and moderately attenuates SuM/MM theta LFPs; it is possible that the PDC results were influenced by the near absence of theta PSD due to the severe attenuation caused by MS-inactivation. Therefore, we examined the validity of our PDC measures when there is a large change of SNR, particularly from HPC LFPs. First, we established the dependency of theta PDC magnitudes on theta SNR by correlating PDC with theta SNR (*r* = 0.36, *p* < 0.001; Figure 8A). By examining the symmetry of PDC spectra at theta frequencies between unmodified data segments and time-reversed segments, we can assess the contribution of SNR on PDC scores. To assess symmetry (see “Materials and Methods” Section for details), difference ratios between HPC->SuM/MM and SuM/MM->HPC PDC scores were computed, and ratios between forward-time and reversed-time data were compared (see Figure 8B for an example); the degree of symmetry indicates vulnerability to artifactual PDC scores due to low SNR (see Figure 8C for examples). Prior to MS inactivation, the difference ratios between forward- and reverse- time PDCs were more asymmetrical (Figure 8D) as indicated by the more scattered distribution of their differences. As expected, where MS inactivation caused changes in a loss of SNR with the theta band, theta PDC difference ratios became more symmetrical, as indicated by a leptokurtic distribution of their differences, evident from the contraction of data points toward the line of identity (Figure 8E). This difference was found to be statistically significant (*t*_(40)_ = 3.28, *p* < 0.003), indicating MS inactivation significantly increased PDC spectra symmetry at theta frequencies, rendering PDC measures calculated post-inactivation questionable. Using a conservative 95% confidence interval computed with PDC difference ratios across both conditions, we extracted data points that had higher PDC asymmetry, hence less susceptible to erroneous PDC measures as a result of SNR issues. Including all data that display asymmetry, there is a statistically non-significant tendency for pre-inactivation PDC to favor a HPC->SuM/MM directionality, and the reversal is true for post-inactivation (*t*_(26)_ = 1.32, *p* = 0.19; Figure 8F). After accounting for data that were deemed to be significantly asymmetric across both epochs, a similar trend exists with only four remaining data points (*t*_(3)_ = 2.78, *p* = 0.07; Figure 8G).

### MS Inactivation Differentially Modulates Overall Afferent Activity and Theta Power in HPC and SuM/MM

**Figure 9:**
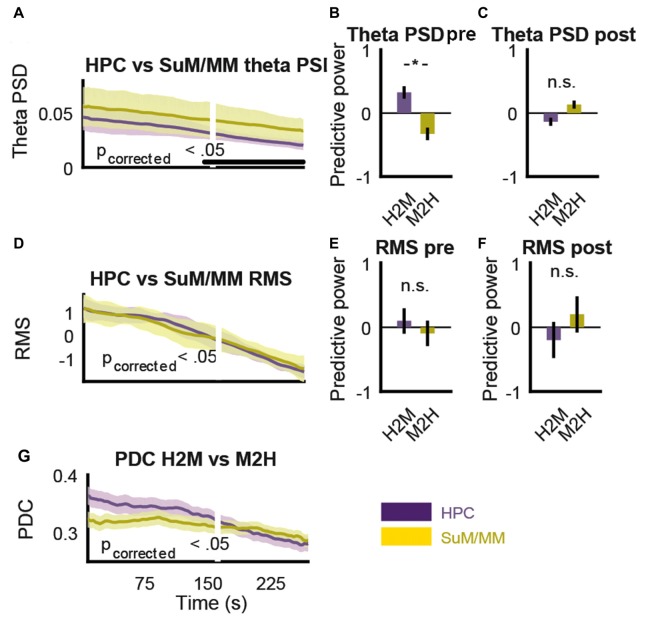
MS-inactivation leads to dissociable changes in non-theta and theta inputs to the SuM/MM. **(A)** Change in HPC and SuM/MM theta PSD across time. The white line denotes the time point where RMS decrease reached 50%. Statistical significance is denoted by the black bar. **(B)** Averaged significant F-ratios from theta PSD regression model (predictive power) before the point of significant theta PSD divergence. **(C)** Averaged significant F-ratios from theta PSD regression model (predictive power) after the point of significant theta PSD divergence. **(D)** Change in HPC and SuM/MM RMS values across time. There is no detected causal influence in either direction. **(E)** Averaged significant F-ratios from RMS regression model (predictive power) before the point of significant theta PSD divergence. **(F)** Averaged significant F-ratios from RMS regression model (predictive power) after the point of significant theta PSD divergence.** (G)** Changes in PDC measures for both directions in the same time period as **(A,D)**. PSD, power spectral density, RMS, root mean squared power; PDC, partial directed coherence; H2M, HPC->SuM/MM PDC score; M2H, SuM/MM->HPC PDC score; HPC, hippocampus; SuM, supramammillary nucleus; MM, mammillary nucleus. *p < 0.05; n.s. not significant.

The purpose of MS inactivation in our study is to attenuate HPC theta, which eliminates its presumed theta rhythmic output to the SuM/MM area. However, the MS projects to both the HPC and SuM/MM (Swanson and Cowan, 1979), so MS inactivation is likely to have a significant impact on both regions beyond theta LFPs. We now ask how much MS contributes to LFPs in all recording sites. Given the partial sensitivity of SuM/MM theta LFPs to MS inactivation, we hypothesized the rate of theta PSD decrease will differ at some point after MS inactivation. Figure 9A shows changes in HPC and SuM/MM theta PSD around the point where a 50% reduction in HPC LFP RMS was detected (white line). Towards the end of the time-course data, there is a decrease in the variance (as indicated by the standard error in shaded areas) of HPC theta PSD, which correlated with a detectable difference (*p* < 0.05, Bonferroni corrected) in theta PSD between the HPC and SuM/MM from this point on. Note that the time point-wise differences in HPC and SuM/MM theta PSD precede the point where HPC LFP RMS reduced by 50%. This point of 50% HPC LFP RMS reduction was in turn preceded by an observable increase in negative slope of HPC theta PSD across time. We then tested the hypothesis that prior to the point of divergence, the decrease in theta PSD rates will be driven by the HPC, and beyond the point of divergence, changes in theta PSD rates should be independent. Figures 9B,C illustrate the differences between estimates of predictive power of theta PSD changes against different recording sites pre (Figure 9B) and post (Figure 9C) the detected point of divergence described in Figure 9A. Prior to the point of theta PSD rate of change deviation, SuM/MM changes appear to be largely driven by HPC changes (*t*_(42)_ = 2.75, *p* < 0.01; Figure 9B) but this interaction reversed in direction with decreased magnitude and became statistically non-significant after the point of deviation (*t*_(42)_ = −1.65, *p* = 0.11; Figure 9C). In contrast, there was no such differentiation when the rates of RMS decrease were plotted against each other (Figure 9D), suggesting a dissociation between the attenuation of total and theta rhythmic input to the HPC and SuM/MM as a result of MS inactivation. Since there were no statistically detectable differences in RMS decreases, we opted to use the same time point, where the theta PSD divergence occurred to examine the dynamics of RMS changes. We found no evidence of preferential HPC or SuM/MM interaction to observed RMS changes (*t*_(42)_ = 0.35, *p* = 0.73, *t*_(42)_ = −0.50, *p* = 0.62; Figures 9E,F, respectively), suggesting MS inactivation equally attenuated the sum of synaptic inputs to the HPC and SuM/MM. Finally, we found no statistical significant differences comparing H2M and M2H PDC estimates in the same time-course data, although the trend suggests the magnitude of M2H direction remains largely constant across time, there is a steady decrease of H2M PDC estimate where the cross-over point coincides with the point of PDS rate of change divergence (Figure 9G).

## Discussion

In this study, we used multi-site LFP recordings, local pharmacological inactivation and Granger causality analysis to dissect the functional interactions between the HPC and the SuM/MM area. Our results support the notion that SuM is involved in HPC frequency coding, particularly at higher end of theta-range oscillations. By using MS inactivation, we were able to show, for the first time, a comparable attenuation but not elimination of theta LFPs in SuM and MM. Theta power attenuation was dissociated from a general attenuation of synaptic inputs, and did not vary along the SuM/MM axis. There was also a tendency for dominant SuM theta frequency to increase after MS-inactivation. Finally, we were unable to detect distinguishing features of theta LFPs recorded in the SuM/MM area prior to or after MS inactivation.

### Methodological Considerations

As mentioned in the “Materials and Methods” Section, the reconstructed recording sites were taken as the mid-distance between the two poles in our twisted bipolar setup to simplify localization and to better reflect the putative source of recorded LFPs. However, variations in bipolar electrode tip separation across rats meant many of the reconstructed recording sites closer to the anatomical boundaries of SuM/MM would in fact be “contaminated” by the adjacent region. This is particularly problematic across the SuM/MM border. Our distance-to-SuM/MM-border metric was designed to ameliorate this caveat, where recordings sites were classified as “more SuM” or “more MM” according to their distance to the anatomical border. This approach also partially addresses the relative undersampling (Figure 1) of MM to allow an establishment of an SuM/MM “gradient” for examining potential differences in theta oscillation characteristics.

It is commonly agreed that LFPs mainly reflect synaptic inputs to the recorded area (Eccles, 1951; Buzsáki et al., 2012), where the relatively slower post-synaptic potentials sum to yield the fluctuation of local ionic balance, thus reflecting local excitability and population output. In our study, theta LFPs are interpreted as a product of summed *input* activity from all potential inputs that subsequently entrains the spiking output of the area (Okun et al., 2010; Denker et al., 2011). However, the use of auto-regressive modeling to determine how much variance can be accounted for between the lagged versions of time-series intrinsically implies each signal representing the output. Therefore, for the purpose of PDC spectra interpretation, LFPs are taken as the surrogate measure of putative entrained oscillatory *output* from the region.

The ability for PDC to provide unbiased estimation of causality may be compromised by a decrease in SNR (Figure 8A), known to contribute to erroneous causality interpretations (Bastos and Schoffelen, 2015). Our extended symmetry analysis between normal-time and reversed-time PDC analysis revealed that the decrease in SNR brought upon by MS inactivation did indeed render most examined signal pairs to yield more symmetrical PDC spectra, indicating PDC estimates reflect the change in SNR rather than directional influence. In this report, we did not seek to statistically determine the relationship between level of PDC (a)symmetry and artifactual causality, as it is beyond the scope of the current study. However, we do note that several studies comparing oscillatory and non-oscillatory states using PDC or related methods may have detected causal influences reflecting the high- and low- SNRs associated with the brain states, respectively, instead of “true” causality (Jackson et al., 2014; Kang et al., 2015; Martínez-Bellver et al., 2017).

### Relative Invariance of Theta Oscillation Profiles across the SuM/MM Area

Based on previous single-cell data with urethane anesthetized rats, it was assumed that the SuM does not require HPC input to maintain theta rhythmic bursting, while the MM is dependent (Kirk et al., 1996). Theta rhythmic spiking activities in the MM also show preferential coupling to HPC CA1 theta oscillations (Kocsis and Vertes, 1994). These findings suggest HPC to be the sole structure responsible for the cell entrainment, and therefore synaptic input driving LFP fluctuations at theta frequencies in the MM. This assumption would suggest that MM theta LFPs should be a direct efference copy of HPC theta LFPs, and different from SuM theta LFPs being a mixture of HPC and extra-HPC theta input. We found a weak association of dominant theta frequency along the SuM/MM axis, which is consistent with indications that SuM may be entrained at a higher theta frequency independent of the HPC. The tendency for theta frequency to be lower in the MM corresponds to the dependency of MM on a frequency-limiting HPC theta rhythmic input demonstrated here. Our statistically non-significant trend of PDC ratios support the assumptions of HPC dependency, where more dorsal (SuM) recording sites have a tendency to better predict HPC theta LFPs, and ventral (MM) sites are better predicted by HPC theta LFPs.

The use of MS inactivation was designed to unequivocally address the issue of HPC-dependency of locally recorded theta LFPs in the SuM/MM. Silencing of the MS-HPC system should abolish HPC theta output, eliminating theta LFPs in the MM and minimally impact SuM theta LFPs. We observed a general decrease of LFP amplitude as a consequence of MS inactivation. The reduction of theta power across all regions appears to be driven by the severe attenuation of HPC theta initially. However, time-course analysis supports the notion that at least one source of theta LFP in SuM/MM persists, where theta power rate of decrease in the SuM/MM area became slower and could no longer be predicted by correlated HPC theta power decrease. The relationship between dominant theta frequency, PDC ratio and our SuM/MM metric became much weaker after MS inactivation. There was also no additional theta-related measurement that provided any predictive value on recording location along the SuM/MM axis after MS inactivation. A lack of any discernible pattern in the anatomical distribution of response types following MS inactivation does not allow the expected (Kirk et al., 1996) distinction of SuM and MM based on theta LFPs. Collectively, current data suggest: (1) MM theta LFPs are not solely dependent on the MS-HPC system in the freely moving rat; (2) there may be at least one source of theta modulation at the Sum/MM that is independent from the MS-HPC system; and (3) HPC theta input to the SuM/MM may interact with other potential theta rhythmic inputs to modulate activity in the SuM/MM area. However, it is at present unclear how much of the changes described above can be attributed to volume conduction from the SuM to the MM (or the reverse) despite our use of bipolar recordings, and how/if extra-HPC theta rhythmic inputs may contribute to theta LFPs recorded from SuM/MM.

### Bi-Directional Frequency Modulation in the HPC-SuM/MM Circuit

As in previous studies based on single-cell recordings in the SuM (Kocsis and Kaminski, 2006), we found theta LFPs recorded from the SuM/MM also appear to bi-directionally interact with HPC theta LFPs in the frequency domain. Superficially, we observed a tendency for HPC theta frequency to be higher compared to a paired SuM/MM recording site. PDC revealed that higher HPC theta frequency is in fact associated with negative PDC HPC-SuM/MM ratio, suggesting SuM input to the HPC drives the faster component of observed dominant frequency in the HPC, consistent with the suggestion that SuM is involved in “accelerating” HPC theta oscillations (Kocsis and Kaminski, 2006). The electrophysiological data are also consistent with lesion data, where SuM (McNaughton et al., 1995) and MM (Sharp and Koester, 2008a) lesions decrease the average HPC theta frequency. Our MS inactivation also indicates that the removal of the MS-HPC system, and presumably all HPC theta rhythmic input to the SuM/MM area, resulted in increases of dominant theta frequency within the SuM/MM area. There is evidence that without the modulating effects of HPC input, SuM intrinsically, or driven by an unknown source, increases its theta rhythmic burst rate (Kirk et al., 1996). It is likely, then, that downstream modulation from the HPC, through the MS/LS is key (Swanson and Cowan, 1979; Leranth and Frotscher, 1989). It is known that local inactivation of the LS, but not the MS by muscimol increases HPC theta frequency (Chee et al., 2015). The increase in HPC theta frequency in this case may be a direct consequence of loss of LS-mediated control over the SuM, allowing theta rhythmic input from the SuM, via the MS, to drive HPC theta at a higher native frequency. A Granger causality analysis on HPC-SuM theta interactions with LS and SuM inactivation can address this possibility. Overall, our freely moving LFP data corroborate SuM single-cell data collected under anesthesia to reinforce the notion that HPC-SuM/MM modulation is indeed bi-directional. Just as the SuM has a role in generating higher frequency theta oscillations in the LFP, the HPC also has a prominent role in slowing theta oscillations in the SuM/MM area. The functional significance and underlying mechanism for this push-pull system is unclear.

### SuM/MM as Sites for Information Integration

We found that MS-inactivation does not fully abolish SuM/MM theta oscillations. In the case of SuM, this is consistent with previous observations (Kirk et al., 1996; McNaughton et al., 2006). In the case of MM, residual LPF oscillations could be simply volume conducted from the SuM but a separate possibility is that there are additional sources of theta-rhythmic input to the MM. The only major afferents that may contribute to theta LFPs in the MM, apart from HPC/EC sources (Meibach and Siegel, 1977; Swanson and Cowan, 1977; Shibata, 1988; Allen and Hopkins, 1989) are within the caudal diencephalon (Allen and Hopkins, 1989; Shibata, 1989) and would include the SuM, and the ventral tegmental nucleus of Gudden (VTNg; Veazey et al., 1982; Hayakawa and Zyo, 1989). Since within the SuM, but not MM, theta burst activity at the cellular level survives MS inactivation (Kirk et al., 1996), SuM efferents probably contribute minimally to direct theta modulation in the MM. Our data cannot pin-point the potential source(s) of MM theta LFPs that survived MS inactivation. Theta LFP and rhythmic bursting activities are present in the VTNg (Bassant and Poindessous-Jazat, 2001; Kocsis et al., 2001) and VTNg may exhibit theta oscillations independent of the HPC in non-anesthetized animals, having a tendency to lead the appearance of HPC theta oscillations (Bassant and Poindessous-Jazat, 2001), thus it is likely theta activity is at least partially independent from the HPC and provides the crucial inhibitory input necessary for theta entrainment of MM neurons. Alternatively, raphe nuclei are known to exhibit theta rhythmicity (Kocsis and Vertes, 1992, 1996; Viana Di Prisco et al., 2002; Kocsis et al., 2006), projecting to both the SuM and MM (Shibata, 1987; Hayakawa et al., 1993; Gonzalo-Ruiz et al., 1999; Vertes et al., 1999) as a potential contributor to extra-HPC theta modulation in the SuM/MM region. Lastly, it should be noted that potential extra-hippocampal input structures to the mammillary area (e.g., VTNg and raphe nuclei) receive input from the MS-HPC system (Swanson and Cowan, 1977, 1979). It is not clear, at present, how this anatomical reciprocity shapes theta frequency interactions in freely moving animals.

The LS-SuM-MM circuit may also play a role. Like the MS, various parts of the LS can oscillate at different theta frequencies simultaneously (Nerad and McNaughton, 2006) and while most show some form of phase-locking to HPC theta, there is a wide range of phase preference (Stewart and Fox, 1990; King et al., 1998; Dragoi et al., 1999). Its diffuse projection pattern and the potential to output theta modulation at various frequencies into the diencephalic region (Swanson and Cowan, 1979; Staiger and Nürnberger, 1991) can account for the varied and seemly randomly distributed theta oscillations in the region reported here and elsewhere (Kowalczyk et al., 2014). In our study, it is unclear if our MS-inactivation also impacted on normal physiology of the LS, and if LS alone is sufficient to mediate downstream modulation from the HPC.

### Functional Considerations

Converging evidence suggests that SuM/MM lesions may modestly reduce ongoing HPC theta frequency while inducing hyperactivity (Pan and McNaughton, 2002; Sharp and Koester, 2008a). This is somewhat paradoxical as HPC theta frequency is linearly related to movement speed (McFarland et al., 1975; Hinman et al., 2011) and vigor (Bland and Vanderwolf, 1972; Whishaw and Vanderwolf, 1973). However, it is known that SuM/MM lesions do differentially affect firing rate-speed relationships across the HPC formation, without affecting spatial representation of HPC place cells (Sharp and Koester, 2008a). The disruption of the HPC theta frequency-movement magnitude relationship by SuM/MM lesions may therefore result from an allocentric and egocentric mis-match of (spatial) context and underlie wider, albeit mild, behavioral deficits mirroring those of HPC lesions (Pan and McNaughton, 2004). A caveat to this is that reciprocal SuM-HPC interactions in a reduced conductance state under anesthesia, where locomotion and sensory functions are very limited, appear to be greater given: (1) HPC dependence on SuM for theta LFPs (Kirk and McNaughton, 1993); (2) apparent SuM dependence on HPC/forebrain for synchronous postictal sharp-wave-like bursting activities (Kirk, 1997); and (3) strong HPC modulation of SuM theta rhythmic bursting rate (Kirk et al., 1996). Currently, in contrast to their cortical counterparts, the generation and behavioral correlates of subcortical theta LFPs are poorly characterized and understood. Our study provides evidence that the presumed SuM-MS-HPC-MM circuit dynamics is much more complex in the freely moving animal, and how the bidirectional interactions may be pertinent to behavioral output has yet to be adequately explored.

The dynamics of theta modulation and behavioral relevance of neuronal activities is patchy at best at the level of SuM/MM. High-density unit and LFP recordings across both structures in freely-behaving animals is necessary to address the discrepancies uncovered between unit activities collected under anesthesia and LFP activities reported here from freely moving rats. Modern circuit dissection tools (Boyden et al., 2005; Nawaratne et al., 2008) should be applied to the SuM/MM-MS/LS-HPC system to elucidate the source(s) of theta modulation, and the outcome of their perturbation. From our data, it is clear that SuM/MM theta LDPs are partially independent from the HPC, and appears to be homogenous across the SuM/MM axis. While brain circuitry involving the MM is far better understood, how patterns of SuM neuronal activity, and its functional correlates/significance is not known. SuM provides independent output to the MS and the HPC itself (Pan and McNaughton, 2004; Vertes, 2015), with the latter targeting less-well understood subregions of DG and CA2. A better understanding of how SuM is involved in modulating HPC activity is crucial for unravelling additional insights into both areas.

Contrary to the predictions made based on single-cell data collected in anesthetized rats, we show theta LFPs are present in the MM independent of HPC in the freely moving rat. Our findings support a fully bi-directional, frequency modulating circuit in HPC-SuM/MM interactions. Data presented here are suggestive of extra-HPC theta input to the SuM/MM, prompting a re-consideration of SuMM/MM being merely relay structures, as already evident from MM lesion studies (Vann, 2013). Identification of local sources of theta LFP with the SuM/MM, and behavioral correlates of SuM single-cell activity are crucial to reveal how the SuM and MM performs local computation and contributes to behavior through coupled theta oscillations with other areas of the brain.

## Author Contributions

MR collected the behavioral, neurophysiological and histological data. CKY carried out part of the histology and analyzed the data. CKY and NM prepared the manuscript and all authors contributed to the conception of the study.

## Conflict of Interest Statement

NM has a confidential disclosure and consulting agreement with Janssen Research & Development, LLC that does not constitute a conflict. The other authors declare that the research was conducted in the absence of any commercial or financial relationships that could be construed as a potential conflict of interest.
